# Virulence and Antimicrobial Resistance of *Listeria monocytogenes* Isolated from Ready-to-Eat Food Products in Romania

**DOI:** 10.3390/microorganisms12050954

**Published:** 2024-05-08

**Authors:** Mihaela Niculina Duma, Laurenţiu Mihai Ciupescu, Sorin Daniel Dan, Oana Lucia Crisan-Reget, Alexandra Tabaran

**Affiliations:** 1 Laboratory of Food Microbiology, Sanitary Veterinary Directorate for Food Safety, 400621 Cluj-Napoca, Romania; dumalsvs@yahoo.co.uk; 2 The Institute of Hygiene and Veterinary Public, The National Sanitary Veterinary Authority for Food Safety, Campul Mosilor 5, 013701 Bucharest, Romania; ciupescu_laurentiu@yahoo.com; 3 Department of Animal Husbandry and Public Health, Faculty of Veterinary Medicine, University of Agricultural Sciences and Veterinary Medicine Cluj-Napoca, 400372 Cluj-Napoca, Romania; sorindan@usamvcluj.ro (S.D.D.); oana.reget@usamvcluj.ro (O.L.C.-R.)

**Keywords:** *Listeria monocytogenes*, food safety, serotyping, virulence genes, antimicrobial resistance

## Abstract

*Listeria monocytogenes* (*L. monocytogenes*) poses a significant threat to food safety due to its ability to cause severe human illness and its resistance to various antibiotics and environmental conditions. This study investigated the prevalence, serotype distribution, virulence gene profiles, and antimicrobial resistance patterns of *L. monocytogenes* in ready-to-eat (RTE) food products from Romania. A total of 8151 samples were analyzed, including various processed dairy, bovine, poultry, pork, and fish products. Bacterial isolation was conducted using the classical standard method, followed by confirmation through biochemical and molecular testing. Among the isolated strains, serotypes 1/2a, 1/2b, and 1/2c were identified, with a prevalence of 75% for serotype 1/2a. Additionally, virulence genes specific to listeriolysin O (*hly*A) and regulatory factor A (*prf*A) were detected in all isolates. Antimicrobial susceptibility testing revealed varying resistance patterns among the *L. monocytogenes* strains. Trimethoprim-sulfamethoxazole and oxacillin showed the highest prevalence of resistance at 26.92% and 23.07%, respectively. However, all strains remained susceptible to ciprofloxacin, levofloxacin, and moxifloxacin. Notably, 23.07% of the isolates exhibited multidrug resistance, with the most common pattern being resistance to oxacillin, penicillin, and tetracycline. Analysis of antimicrobial resistance genes identified tetracycline resistance genes, particularly *tet*(C), *tet*(M), and *tet*(K), in a significant proportion of isolates. The presence of *amp*C and *dfr*D genes was also notable, indicating potential mechanisms of resistance. These results emphasize the necessity for ongoing surveillance of *L. monocytogenes* in RTE foods and emphasize the importance of thorough monitoring of antimicrobial resistance to guide public health strategies within the European Union.

## 1. Introduction

Listerias, a group of bacteria within the Clostridium phylogenetic branch and Listeriaceae family, comprises a genus with 26 species, including 18 *Listeria* species recognized and described for the first time in 2009. Based on genetic and observable traits, a distinct group of six species, known as Listeria sensu strictu, share common features like thriving in low temperatures and having flagellar motility, with the pathogenic *Listeria monocytogenes* being part of this group. The remaining species, termed Listeria sensu lato, form three separate phylogenetic groups, suggesting they could be recognized as distinct genera. These proposed genera typically lack pathogenicity, are often non-motile (except for *Listeria grayi*), possess nitrate reduction abilities (except *Listeria floridensis*), and yield negative results in the Voges–Proskauer test (except *L. grayi*) [1].

Human pathogens are of particular concern to health authorities. While the incidence of listeriosis is lower compared to other foodborne zoonoses, it remains worrisome due to its severity and resilience, making eradication challenging [2]. Various strains are resistant to multiple classes of antibiotics, and many *L. monocytogenes* strains can withstand chemical treatments, such as disinfectants [3]. Consequently, combating *L. monocytogenes* contamination in food processing facilities is difficult, as it can form biofilms on various surfaces and survive and multiply under extreme environmental conditions, such as low temperatures, wide pH ranges, and high salt concentrations [4].

The rare but severe consequences of human food contamination by *L. monocytogenes*, and to a lesser extent by *L. ivanovii*, justify the implementation of monitoring these bacteria in the main food processing sectors, particularly in animal-derived products [5].

The pathogenicity of *L. monocytogenes* is attributed to the invasion of host cells by several virulence factors. Numerous studies highlight the role of extracellular listeriolysin O, which regulates host cell functions [6]. This pore-forming protein, encoded by the *hly* gene, is crucial for the bacterium’s interaction with the host cell. Other virulence-associated genes include *act*A, responsible for actin polymerization and mobility, *plc*A and *plc*B, involved in membrane lysis during cell propagation, and *inl*A, facilitating bacterial invasion into intestinal epithelial cells [7,8].

Surveillance of Listeria’s presence is crucial because it allows, among others, the identification and characterization of specific serotypes, such as 1/2a, 1/2b, and 4b, which are responsible for the majority of human listeriosis cases [9].

In 2022, listeriosis ranked as the fifth most frequently reported zoonosis in the EU, with 2738 cases—a 15.9% increase in the EU notification rate compared to 2021 [10].

*Listeria* spp. typically demonstrate susceptibility to a broad array of antimicrobials; however, the emergence of the first multi-resistant *L. monocytogenes* strain dates back to 1988 [11]. Since then, instances of antibiotic-resistant *L. monocytogenes* isolates have been identified in food, environmental, and human listeriosis cases [11]. Currently, the recommended therapy for human listeriosis involves a combination of a β-lactam antibiotic (such as ampicillin or penicillin) with an aminoglycoside (such as gentamicin), while alternative treatment options include vancomycin, erythromycin, and trimethoprim-sulfamethoxazole for pregnant women or patients with β-lactam allergies [12]. The prevalence and patterns of resistance are influenced by antibiotic utilization practices and regional disparities. Hence, the investigation and surveillance of *Listeria* spp. antibiotic susceptibility across different geographical regions is crucial for safeguarding public health [13]. Due to these factors, *L. monocytogenes* represents a considerable threat to the food sector, notably RTE food manufacturers, with meat products remaining among the top three RTE food categories commonly linked to human listeriosis [14].

The culinary practices in Romania are characterized by a strong inclination towards consuming raw or undercooked pork delicacies like ham, sausages, bacon, rillettes, and marinated loin, often prepared at home using fresh meat purchased from retail outlets. These traditional methods of pork consumption carry a heightened risk of contamination with foodborne pathogens. Multidrug-resistant strains of *L. monocytogenes*, for instance, can potentially infiltrate the food supply chain via raw meat products, posing a significant threat of severe illness in humans. An investigation into antimicrobial susceptibility was conducted on 26 *L. monocytogenes* isolates obtained from severe clinical cases of listeriosis in Romania [15]. The study revealed a notable proportion (82%) of isolates exhibiting resistance to at least one antibiotic, though no instances of multidrug resistance were observed. Notably, 18 of the examined strains displayed resistance to ciprofloxacin [15]. However, the authors of the conducted study [15] acknowledged the methodological constraint of the investigation, particularly the absence of serotyping of the *L. monocytogenes* isolates. As a result, critical questions regarding the prevalence and distribution of clinically relevant serotypes, such as 1/2a, 1/2b, 1/2c, and 4b, remained unanswered. Therefore, there is a lack of updated data on the prevalence of *L. monocytogenes* in Romanian RTE food products and the characterization of virulence, serotype, and antimicrobial resistance. This study aimed to assess the presence of *L. monocytogenes* in various food matrices collected in the northwestern region of Romania and characterize the virulence, serotype, and antibiotic susceptibility profiles of isolated strains using classical and molecular methods.

## 2. Materials and Methods

### 2.1. Sample Collection

The research involved examining 8151 RTE food items received by the Sanitary Veterinary Food Safety Laboratory from the northwestern region of Romania. These samples were analyzed to verify compliance with safety standards, specifically the absence of *Listeria* spp. in RTE meat products. The samples tested in this food safety laboratory encompassed processed dairy products, bovine meat products, poultry meat products, processed pork meat (such as sausages, ham, and bacon), and fish products. The study comprised the period 2019–2022, during which time the following number of samples were tested: 2019—n = 2178; 2020—n = 2009; 2021—n = 1930; 2022—n = 2034. All the samples were RTE meat products brought directly to the laboratory by the producers in their natural package and in refrigerated storage conditions (0–4 °C).

### 2.2. Bacterial Isolation

The bacterial isolation protocol was performed according to the horizontal detection and counting method for *L. monocytogenes* [16]. Briefly, 25 g of each sample investigated was inoculated in 225 mL of selective supplement half Fraser broth (Sharlau, Sentmenat, Spain) and then incubated for 25 ± 1 h at 30 ± 1 °C. Afterward, a second enrichment was performed, which consisted of adding 0.1 mL of the broth culture in 10 mL of full-strength Fraser broth (UVM II Selective Supplement Scharlau/Spain) and incubation at 37 °C for 24 ± 2 h. A loopful of each of the half-and full-strength Fraser broths was plated on the chromogenic agar ALOA (Scharlau/Spain) and Oxford agar (Merck, Darmstadt, Germany). All the plates were incubated in aerobic conditions at 37 °C for 24–48 h.

Specific colonies developed on ALOA and Oxford agar were then re-streaked on tryptic soy agar supplemented with 0.6% yeast extract (TSA-YE) (BioLife, Monza, Italy). The incubation was performed at 37 °C for 24 h. The colonies developed on TSA-YE media were confirmed by Gram Staining, Hemolysis tests on blood agar (Columbia Blood Agar Base Oxoid/Basingstoke, UK + Defribinated Horse Blood), carbohydrate utilization test (Carbohydrates Utilisation Broth Base ISO Condalab/Madrid, Spain), CAMP test (reference strain *Staphylococcus aureus* ATCC 6538P lot 827-392-3; *Rodococcus equi* ATCC 6939 lot 697-78-6; *L. monocytogenes* ATCC 13932 lot 129-101-81; *L. ivanovii* ATCC 19119 lot 815-66-5; *L. innocua* ATCC 33090 lot 814-182-2). The confirmation of *Listeria monocytogenes* was also performed by biochemical testing on the VITEK 2 GP Immunodiagnostic Assay System (Biomerieux/Craponne, France) according to the manufacturer’s instructions.

### 2.3. Susceptibility Testing

After isolation, the frozen *L. monocytogenes* strains were thawed and then plated onto Brain Heart Infusion agar (Merck, Germany) before being incubated at 37 °C for 24 h. For antibiotic susceptibility testing, the disc diffusion method was applied, following the standard protocol recommended by the European Committee on Antimicrobial Susceptibility Testing (EUCAST). Briefly, 5–6 colonies from overnight cultures were suspended in 1 mL of 0.9% NaCl solution, and the turbidity was adjusted to a 0.5 McFarland standard. Afterwards, the suspension was inoculated onto Mueller–Hinton agar (Merck, Germany). Antibiotic discs were placed on the agar surface at intervals of 3 cm, each disc containing a specific antibiotic concentration: ampicillin (10 μg), cephalothin (30 μg), ciprofloxacin (5 μg), clindamycin (2 μg), chloramphenicol (30 μg), gentamicin (10 μg), levofloxacin (5 μg), moxifloxacin (5 μg), meticillin (5 μg), oxacillin (1 μg), penicillin G (10 μg), rifampicin (30 μg), trimethoprim-sulfamethoxazole (1.5/23.5 μg), tetracycline (30 μg), and vancomycin (30 μg). Subsequently, the plates were incubated at 37 °C for 24 h. The diameter of the inhibition zones was measured to the nearest millimeter. Interpretation of the data was conducted following the EUCAST criteria [17]. In cases where the EUCAST guidelines did not provide resistance criteria for *Listeria*, any missing breakpoints were supplemented with those recommended for *Staphylococcus aureus* and *Enterococcus* spp. according to CLSI standards [18].

### 2.4. DNA Extraction from Colonies

The bacterial DNA was extracted using InstaGene Matrix (BIO-RAD, 732-6030, South Granville, Australia), according to the manufacturer protocol, with few modifications. Briefly, two colonies were resuspended in 150 µL of 6% *w*/*v* Chelex resin solution (Merck, Germany), followed by incubation at 56 °C for 20 min and 1500 vibrations/min on thermomixer and then the tubes were placed at 98 °C for 15 min. The samples were later centrifuged at 12,000 rpm for 5 min, and the supernatant was stored at −18 °C until use.

### 2.5. Polymerase Chain Reaction (PCR) for Serotype Testing

The multiplex PCR was performed through the use of primers described by D’Agostino et al. (2004) [19] and Doumith et al. (2004) [20] (Table S1—Supplementary File). The 25 µL PCR reaction consisted of 2.5 µL bacterial DNA, 12.5 µL of 2× QIAGEN Multiplex PCR Master Mix (Qiagen, Hilden, Germany), and 10 µL of primers mix formed by 0.4 µM (forward and reverse primers) for lmo1118, 0.4 µM for lmo0737, orf 2819 and orf 2110, 0.1 µM for *prs* and 0.2 µM for *prf*A. The multiplex PCR comprised a step for pre-denaturation and polymerase activation at 95 °C for 5 min, a step of 40 amplification cycles (denaturation at 95 °C for 20 s, hybridization at 54 °C for 40 s, and elongation at 72 °C for 90 s) followed by a final step at 72 °C for 7 min for elongation. The electrophoresis gel was prepared from 2% agarose (Bioline, London, UK) in TAE (Bioline, UK). Electrophoresis was performed for 1 h and 30 min at 100 V, using 10 µL of DNA ladder (Promega, Southampton, UK) and 10 µL of amplicons, all of them mixed with 2 µL of 6x dual-action nontoxic fluorescent nucleic acid stain and loading dye (RedSafe, Bioline, UK). The expected bands for each gene identification are detailed in Table S1 (Supplementary File).

### 2.6. Polymerase Chain Reaction (PCR) for Virulence Testing

For virulence testing, a multiplex PCR was performed through the use of primers described in Table S2 (Supplementary File). The protocol followed the steps previously described for serotype testing. The *L. monocytogenes* strain ATCC 19112 was used as a positive reference for the interpretation in the PCR method.

### 2.7. Polymerase Chain Reaction (PCR) for Antimicrobial Resistance Genes Testing

The molecular method was used to detect the genes conferring streptomycin, chloramphenicol, trimethoprim, ß-lactam, and tetracycline resistance. Primers and temperature conditions for amplification are shown in Table S3 (Supplementary File).

### 2.8. Statistical Analysis

The statistical analysis was conducted using OriginPro 8.5 software (OriginLab Corporation, Northampton, MA, USA) for the Chi-squared test. A significance level of *p* ≤ 0.05 was applied for result interpretation. Additionally, the Tukey post hoc test was used to evaluate the statistically significant variances between the resistant and intermediate-resistant *L. monocytogenes* isolates.

## 3. Results

### 3.1. Prevalence, Serotype, and Virulence of L. monocytogenes in the RTE Food Products

Twenty-six isolates of *L. monocytogenes* were found from the total number of samples investigated in this study (n = 8151). The RTE food products that tested positive were mainly originating from pork meat and fish meat. Taking into consideration the timeframe in which they were isolated, we found that the majority of the strains (n = 21) were isolated during the year 2022. In 2019 and 2021, only one sample was found positive for the presence of *L. monocytogenes*, while in 2020, three samples were positive. The difference in the number of isolates cannot be attributed to the number of samples investigated, taking into account that in each year, the approximately same number (n = 2178, 2019; n = 2009, 2020; n = 1930, 2021; n = 2034, 2022) was taken into examination. Isolates were assigned to serotypes 1/2a, 1/2b, and 1/2c, with a higher prevalence for 1/2a serotypes (75%). Six serogroup determinants were investigated in our study, *prs* being the most prevalent and detected in all samples. *prf*A was also a characteristic virulence gene in all the samples belonging to the 1/2a serotype. Two of the 1/2a serotype isolates tested positive also for *orf* 2819 (Figure S1, Supplementary File).

A total number of five serotypes were detected in this study (Figure S1, Supplementary File) that had a distinctive pattern of virulence also. All the isolates tested positive for the virulence genes specific to listeriolysin O (*hlyA*) and for the gene-specific regulatory factor A (*prf*A) (Table 1, Figure S1 Supplementary File). We found that all of the strains isolated in 2022 (n = 21) tested positive for all the virulence genes investigated. The strain isolated in 2021 also showed positivity to the internalin (*inl*J) gene, while the strains isolated in 2019 and 2020 tested positive only for LIPI-1 genes (*prf*A and *hly*A).

### 3.2. Susceptibility to Antibiotics in L. monocytogenes Isolated in RTE Products

This study aimed to assess the antibiotic resistance profiles of 26 *L. monocytogenes* strains isolated from RTE products available in the Romanian market. Among the antibiotics tested, trimethoprim-sulfamethoxazole exhibited the highest prevalence of resistance (26.92%), followed by oxacillin (23.07%) (Table 2). Interestingly, all tested strains showed susceptibility to ciprofloxacin, levofloxacin, and moxifloxacin. Additionally, intermediate resistance was observed in a minority of strains to ampicillin (7.69%), meticillin (3.94%), penicillin G (3.94%), and oxacillin (23.07%). The statistical analysis demonstrated significant differences (*p* < 0.01) in prevalence between the resistant strain isolates and the intermediate ones.

It is important to note that intermediate resistance indicates that while the antibiotic may still have efficacy, its dosage might need adjustment for optimal effectiveness. Notably, 23.07% of the *L. monocytogenes* strains exhibited multidrug resistance (MDR), defined as resistance to three or more antibiotics. The most prevalent multidrug resistance pattern observed was resistance to oxacillin, penicillin, and tetracycline (Table 3).

### 3.3. Antimicrobial Resistance Gene Presence in L. monocytogenes

All *L. monocytogenes* isolates exhibited the presence of various antimicrobial resistance gene sequences. The predominant resistance gene was associated with tetracycline, with twenty out of 26 isolates testing positive for at least one of the relevant genes. The *tet*(C) genetic determinant was identified in 63% of tetracycline-resistant isolates, followed by *tet*(M) (42%) and *tet*(K) (42%). Conversely, other tetracycline resistance genes (*tet*(A), *tet*(B), *tet*(L), and *tet*(S) were absent in all tested *L. monocytogenes* isolates from the food products. None of the isolates tested positive for *aad*6 and *cat* genes; however, a considerable proportion of *L. monocytogenes* isolates were positive for *amp*C (58%) and *dfr*D (50%) (Table 1).

## 4. Discussion

A study published by EFSA in 2013 and carried out between 2010 and 2011 showed that the prevalence of *L. monocytogenes* varied among different categories of RTE food sampled across the European Union. Specifically, the prevalence rates at the end of the shelf-life were 10.3% for smoked or graved fish, 2.07% for packaged heat-treated meat products, and 0.47% for soft or semi-soft cheese products [21]. Our research has shown that the prevalence in Romania during our four-year interval (2019–2022) is 0.31% in the RTE products investigated. The highest prevalence of the total number found positive (n = 26) was in pork meat-originating products (42.3%), and the highest number of positive samples was seen in 2022 (n = 21). It is plausible that the observed increase in positive samples for *L. monocytogenes* in 2022 could be attributed to various factors, including the impact of the COVID-19 pandemic and changes in production and consumption patterns post-pandemic. During the COVID-19 pandemic, there were disruptions in food production and distribution systems due to lockdowns, restrictions on movement, and workforce shortages. These disruptions could have affected food safety measures and increased the likelihood of contamination or cross-contamination in food processing facilities. Additionally, changes in consumer behavior, such as increased reliance on RTE foods or shifts in dietary preferences, might have influenced the prevalence of *L. monocytogenes* contamination in certain food products. However, without specific data on the impact of the COVID-19 pandemic on food safety practices and *L. monocytogenes* contamination rates, it is challenging to definitively attribute the increase in positive samples solely to pandemic-related factors.

In 2011, a separate investigation assessing the prevalence of *Listeria* spp. in foodstuffs from the southern region of Romania demonstrated a higher prevalence for *L. innocua* compared to *L. monocytogenes* [22]. However, it highlights the increase in the contamination of foodstuffs by *L. monocytogenes* and the resulting significant economic problem, specifying that the presence of strains of *Listeria* spp. proved to be a useful indicator during all stages of the food production chain [23,24,25,26,27]. Between 2016 and 2019, Romania registered increasing trends in human listeriosis cases, with nine confirmed cases in 2016 and 17 cases in 2019 [14]. However, data concerning the prevalence and antibiotic resistance profiles of strains isolated from RTE products in Romania are lacking. The increased prevalence of *L. monocytogenes* isolates in 2022 compared to previous years suggests a potential need for heightened surveillance and control measures to prevent further contamination and mitigate public health risks.

Coroneo et al. (2016) [28] documented similar findings in *L. monocytogenes* isolates from cheese in Italy, observing variable rates of virulence gene detection. Furthermore, other investigations have reported comparable results in samples obtained from various food sources, including raw milk, milking equipment, worker’s hands, and clinical specimens [29]. Similar to our findings, other research revealed the presence of virulence genes in all examined *L. monocytogenes* isolates isolated from diverse food types [30,31,32,33]. It is recognized that certain polymorphisms and point mutations present in specific virulence genes may contribute to attenuated virulence in *L. monocytogenes* strains [34,35]. Consequently, the absence or presence of virulence factors could serve as a tool not only to assess risks associated with food consumption but also those linked to strain-specific virulence parameters of *L. monocytogenes* [36]. Our results revealed that twenty-one strains tested positive for all investigated virulence factors (Table 1). The pathogenicity of *L. monocytogenes* is governed by several virulence factors, notably the family of internalins, which are bacterial surface proteins responsible for the internalization (entry) of *L. monocytogenes* into cells (*Inl*A, *Inl*B) [37], and dissemination between cells (*Inl*C) [38]. Another critical factor is listeriolysin O (LLO), a protein facilitating the survival and intracellular multiplication of this pathogen by enabling bacterial escape from the phagosome into the cytoplasm of infected host cells [39].

The identified virulence factors in the context of public health risk underscore the potential severity of *L. monocytogenes* infections associated with RTE food products, particularly those originating from pork and fish meat. The predominance of serotype 1/2a, which accounted for 75% of isolates, suggests a higher propensity for virulence among these strains. Furthermore, the detection of key virulence genes such as listeriolysin O (*hly*A), regulatory factor A (*prf*A), and internalin (*inl*J) across multiple serotypes highlights the widespread presence of virulence determinants capable of facilitating host cell invasion and evasion of immune responses.

Of particular concern is the consistent detection of these virulence genes among strains isolated across different years, with all strains isolated in 2022 testing positive for all virulence genes investigated. This suggests a persistent risk of severe *L. monocytogenes* infections associated with RTE food consumption over time. Additionally, the identification of unique virulence patterns among different serotypes underscores the complexity of *L. monocytogenes* pathogenesis and the need for comprehensive surveillance and control measures to mitigate public health risks associated with these pathogens. Overall, the presence of virulence factors such as *hly*A, *prf*A, and *inl*J in *L. monocytogenes* isolates from RTE food products underscores the potential for severe illness in case of contamination.

The current investigation revealed a notable resistance among *L. monocytogenes* to ampicillin and penicillin, which are commonly used in the treatment of listeriosis. Similar observations of ampicillin and penicillin resistance have been documented in various turkey and chicken meat samples by several researchers [40,41,42].

Conversely, some studies have reported a high susceptibility of *L. monocytogenes* isolates to these antibiotics [43]. In our study, we found a relatively high proportion of isolates that also exhibited resistance to oxacillin (23.07%). Similarly, in Italy, Pesavento et al. (2010) [44] found a high prevalence of *Listeria* spp. isolates resistant to methicillin and oxacillin. It is noteworthy that *Listeria* spp. can transmit or acquire methicillin resistance genes from *Enterococcus* spp., given that methicillin is commonly employed in the treatment of *Enterococcus* infections. The antibiotic susceptibility profiles of *L. monocytogenes* isolates in our study, particularly the high prevalence of resistance to trimethoprim-sulfamethoxazole and oxacillin, have implications for clinical management and treatment strategies for listeriosis cases. Also, the susceptibility of the isolates to certain antibiotics such as ciprofloxacin, levofloxacin, and moxifloxacin provides valuable information for clinicians in selecting appropriate antimicrobial therapy for listeriosis infections, especially in cases of severe illness or systemic involvement.

*L. monocytogenes* isolated from RTE products found in Romania demonstrated sensitivity to fluoroquinolones (ciprofloxacin, levofloxacin, and moxifloxacin). Compared to other studies, our isolates showed great sensitivity to fluoroquinolones, while others demonstrated a high rate of antimicrobial resistance to fluoroquinolones and tetracycline [42]. The sensitivity of *L. monocytogenes* isolates to antibiotics commonly used in the treatment of listeriosis (rifampicin, gentamicin, and clindamycin) was to that exhibited in previous studies [42,44]. Chloramphenicol resistance was observed in a small percentage of *L. monocytogenes* (3.84%). Similar findings have been reported in other studies that were focused on areas where the resistance to this antibiotic may be attributed to its illegal use in veterinary medicine [42]. Moreover, a slightly concerning resistance was noticed to cephalothin (15.38%) which is somehow consistent with previous reports suggesting natural resistance to cephalosporins in *Listeria* spp. [44].

The prevalence of antibiotic resistance among *Listeria* spp. has garnered increased attention in recent years. A lot of recent studies have focused on this subject [41,42,44], revealing both multiple resistant strains but also susceptibility to various classes [44], such as gentamicin and chloramphenicol. Our study also revealed a low rate of resistance to gentamicin and chloramphenicol (3.84%). Previous studies [40,42,43] also reported varying percentages of ampicillin-resistant *L. monocytogenes* strains. Compared to these studies, ours has proved a higher percentage of resistance to ampicillin among *L. monocytogenes* isolates (19.23%). Our findings suggest an increasing trend in multiresistance among *Listeria* isolates in RTE products found in Romania.

Resistance among *L. monocytogenes* isolates was not concentrated according to food products or origin but rather distributed randomly among various types of food products. The presence of a high number of samples positive for resistance genes to tetracycline and beta-lactams, both critical antimicrobial agents in veterinary medicine [45], is concerning. Despite this resistance, *L. monocytogenes* remains susceptible to primary drugs recommended for treating Listeriosis according to CLSI guidelines [46]. When comparing the results with the ones from the disk diffusion test, we observed disparities between the presence of resistance genes and the phenotypic expression of antibiotic resistance in the tested samples. The results obtained from classical antibiogram testing, which assesses antibiotic susceptibility based on bacterial growth inhibition, did not consistently align with the presence of specific resistance genes. Specifically, there was an obvious difference between the presence of the tetracycline (TET) resistance gene and the actual resistance observed in the samples. While a high percentage (76.92%) of the samples tested positive for the TET resistance gene, only 19.23% exhibited phenotypic resistance to tetracycline. This indicates that although the genetic determinant for tetracycline resistance was prevalent in the samples, the bacteria did not necessarily display resistance when exposed to tetracycline in the laboratory setting. Also, there was a similar discordance in the case of ampicillin resistance. Despite a relatively low number of samples showing phenotypic resistance to ampicillin (only five samples), a substantial proportion (58%) tested positive for the *amp*C resistance gene associated with ampicillin resistance. These findings indicate the complexity of antibiotic resistance mechanisms in bacteria. The presence of resistance genes does not always guarantee phenotypic resistance, and vice versa. Various factors, including gene regulation, expression levels, and interactions with other genetic elements, can influence the manifestation of antibiotic resistance in bacterial populations. Further research is needed to elucidate the underlying mechanisms driving these discrepancies and their implications for antibiotic resistance surveillance and management strategies [47]. The presence of antimicrobial resistance genes, particularly those associated with tetracycline resistance, highlights the importance of judicious antibiotic use and ongoing surveillance of antimicrobial resistance in *L. monocytogenes* strains to inform clinical decision-making and ensure effective treatment outcomes.

The heightened resistance to various classes of antibiotics observed in our study could be attributed to the extensive use of animal feed additives and veterinary treatments, which also relates to isolates found in various types of animal-origin food products [48]. Processing procedures during farming practices, slaughtering, and transportation are also critical factors that may contribute to antibiotic resistance in food [49]. Over time, bacteria like *Listeria* have demonstrated the ability to develop resistance mechanisms through the acquisition of genetic materials from other bacterial species, contributing to the concerning rise in antimicrobial resistance. These findings underscore the urgent need for effective strategies to address antimicrobial resistance and ensure the successful treatment of infectious diseases.

## 5. Conclusions

This study provides valuable insights into the prevalence, virulence factors, and antibiotic resistance profiles of *L. monocytogenes* strains isolated from RTE products in the Romanian market. Distinct serotypes among *L. monocytogenes* isolates were revealed, each with specific virulence gene patterns. The findings reveal concerning levels of resistance, with trimethoprim-sulfamethoxazole and oxacillin showing the highest prevalence of resistance among the tested antibiotics. Conversely, the strains exhibited susceptibility to ciprofloxacin, levofloxacin, and moxifloxacin, indicating potential treatment options for listeriosis infections. Of particular concern is the identification of multidrug resistance in a notable proportion of strains, highlighting the potential challenge in managing infections caused by these bacteria. Additionally, all isolates exhibited multiple antimicrobial resistance genes, with tetracycline resistance genes being the most prevalent. These findings underscore the importance of ongoing surveillance and monitoring of antibiotic resistance in foodborne pathogens to inform public health strategies and combat the emergence of multidrug-resistant strains.

## Figures and Tables

**Table 1:** Antimicrobial resistance genes and virulence characteristics of all strains found in the study.

No of Sample/Year	Source	Serotype	Virulence Gene Detected	Antimicrobial Resistance Genes Detected
896, 897, 898, 899, 900, 901/2022	Pork meat bacon	1/2a-3a	*hly*A, *plc*B, *act*A, *prf*A, *inl*J	*Tet*(M), *am*pC
669/2019	Pork meat sausages	1/2a-3c	*hly*A, *prf*A,	*Tet*(M), *Tet*(C), *Tet*(K), *dfr*D
1018, 1019/2020	Fish fillet	1/2a-3a	*hly*A, *prf*A,	*am*pC, *dfr*D
830/2020	Trout fish butter creme	1/2a-3a	*hly*A, *prf*A,	*Tet*(M), *amp*C
102, 103, 104, 105, 106, 107/2022	Poultry meat salad	1/2a-3a	*hly*A, *plc*B, *ac*tA, *prf*A, *inl*J	*Tet*(C), *Tet*(K), *dfr*D
93, 94, 95, 96/2022	Pork meat hotdog	1/2a-3a	*hly*A, *plc*B, *ac*tA, *prf*A, *inl*J	*amp*C, *dfr*D
2018, 2019, 2020, 2021, 2022/2022	Beef meat salad	1/2b	*hly*A, *plc*B, *ac*tA, *prf*A, *inl*J	*Tet*(C), *Tet*(M), *Tet*(K)
330/2021	Raw milk cheese	1/2b	*hly*A, *prf*A, *inl*J	*Tet*(K), *amp*C

**Table 2:** Antibiotic susceptibility of the *L. monocytogenes* isolates from RTE food products in Romania.

Antimicrobial	No of Resistant *L.monocytoegenes* Isolate (%)	No of Intermediate Resistant *L.monocytoegenes* Isolate (%)
Ampicillin	5 (19.23)	2 (7.69%)
Cephalothin	4 (15.38%)	0
Ciprofloxacin	0	0
Clindamycin	1 (3.84%)	0
Chloramphenicol	1 (3.84%)	0
Gentamicin	1 (3.84%)	0
Levofloxacin	0	0
Moxifloxacin	0	0
Meticillin	2 (7.69%)	1 (3.84%)
Oxacillin	6 (23.07%)	2 (7.69%)
Penicillin G	4 (15.38%)	1 (3.84%)
Rifampicin	1 (3.84%)	0
Trimethoprime-sulfamethoxazole	7 (26.92)	1 (3.84%)
Tetracycline	5 (19.23)	0

**Table 3:** Resistance patterns in *L. monocytogenes* isolated from RTE products.

Multiple Resistance Pattern	Origin of Strain	Resistance Pattern	No. of Isolates (%)
One type of antimicrobial	Fish fillet	SMX	3 (11.53%)
Two types of antimicrobials	Pork meat	SMX, OXA	2 (7.69%)
	Trout fish	AMP, CEPH	2 (7.69%)
	Beef meat	MET, TET	1 (3.84)
	Poultry meat	PEN, GEN	1 (3.84)
	Pork meat	OXA, CHL	1 (3.84)
Three types of antimicrobials	Beef meat	AMP, CEPH, TET	1 (3.84)
	Pork meat	AMP, SMX, PEN	1 (3.84)
	Pork meat	CLIN, MET, OXA	1 (3.84)
		OXA, PEN, TET	1 (3.84)
Four types of antimicrobials	Beef meat	SMX, CEPH, RIF, TET	1 (3.84)
	Poultry meat	AMP, OXA, PEN, TET	1 (3.84)

## Data Availability

The data presented in this study are available on request from the corresponding author due to privacy.

## Supplementary Materials

The following supporting information can be downloaded at https://www.mdpi.com/article/10.3390/microorganisms12050954/s1, Figure S1: PCR profile for virulence gene detection; Table S1: Target genes for serogroup, expected size of amplicons and primers used; Table S2: Target genes for antibiotic resistance, expected size of amplicons and primers used. Table S3: Primer sequence, expected product size, and PCR annealing temperature used for amplification.
