# Supplementary material for: Virulence and Antimicrobial Resistance of Listeria monocytogenes Isolated from Ready-to-Eat Food Products in Romania
Source: Microorganisms. 2024 May 8;12(5):954. doi: 10.3390/microorganisms12050954 (PMC11123701; doi:10.3390/microorganisms12050954)
Supplement: Supplementary file 1 [file microorganisms-12-00954-s001.zip › microorganisms-2941892-supplementary.pdf]

Table S1: Target genes for serogroup, expected size of amplicons and primers used

Target gene	Size (bp)	Primer sequence (5′-3′)	Molecular group					Reference
			**IIa**	**IIb**	**IIc**	**VIa**	**VIb**	
prfA	274	GATACAGAAACATCGGTTGGC<br>GTGTAATCTTGATGCCATCAGG	+	+	+	+	+	11
prs	370	GCT GAA GAG ATT GCG AAA GAA G<br>CAA AGA AAC CTT GGA TTT GCG G	+	+	+	+	+	12
orf 2819	471	AGC AAA ATG CCA AAA CTC GT<br>CAT CAC TAA AGC CTC CCA TTG	-	+	-	-	+	
Orf 2110	597	AGT GGA CAA TTG ATT GGT GAA<br>CAT CCA TCC CTT ACT TTG GAC	-	-	-	-	+	
lmo0737	691	AGG GCT TCA AGG ACT TAC CC<br>ACG ATT TCT GCT TGC CAT TC	+	-	+	-	-	
lmo 1118	906	AGG GGT CTT AAA TCC TGG AA<br>CGG CTT GTT CGG CAT ACT TA	-	-	+	-	-	

Table S2: Target genes for virulence testing, expected size of amplicons and primers used

Target gene	Size (bp)	Primer sequence (5′-3′)	Amplification condition	Reference
inlJ	238	F-TGTAACCCCGCTTACACAGTT<br>R-AGCGGCTTGGCAGTCTAATA	95°C/5min<br>94°C/80s<br>58°C/90s<br>72°C/6min	13
actA	650	F-CCAAGCGAGGTAAATACGGGA<br>R-GTCCGAAGCATTTACCTCTTC		
prfA	467	F-ACCAATGGGATCCACAAGA<br>R-CAGCTGAGCTATGTGCGAT		
hlyA	404	F-ATCATCGACGGCAACCTCGGAGAC<br>R-CACCATTCCCAAGCTAAACCAGTGC		
plcB	289	F-AATATTTCAATCAATCGGTGGCTGA<br>R-GGGTAGTCCGCTTTCGCTCTT		

Table S3: Primer sequence, expected product size and PCR annealing temperature used for the amplification of resistance genes.

Targeted gene	Expected size (bp)	Primer sequence	Annealing temperature (°C)	Targeted antibiotic class
aad6	978	F: AGAAGATGTAATAATATAG	56	Streptomycin
		R: CTGTAATCACTGTTCCCGCCT		
cat	384	F: GAACAGGAATTAATAGTGAG	54	Chloramphenicol
		R: GGTAACCATCACATAC		
dfrD	199	F: AGAGTAATCGGCAAGGATAACG	56	Trimethoprim
		R: AATGGGCAATTTCACAATCC		
ampC	550	F: TTCTATCAAMACTGGCARCC	60	Beta-lactams
		R: CCYTTTTATGTACCCAYGA		
*bla*TEM	692	F: TTTCGTGTCGCCCTTATTCC		
		R: CCGGCTCCAGATTTATCAGC		
*bla*Z	173	F: ACTTCAACACCTGCTGCTTTC		
		R: TGACCACTTTTATCAGCAACC		
Tet(A)	210	F: GCTACATCCTGCTTGCCTTC	55	Tetracycline
		R: CATAGATCGCCGTGAAGAGG		
Tet(B)	659	F: TTGGTTAGGGGCAAGTTTTG		
		R: GTAATGGGCCAATAACACCG		
Tet(C)	418	F: CTTGAGAGCCTTCAACCCAG		
		R: ATGGTCGTCATCTACCTGCC		
Tet(K)	361	F: GTAGCGACAATAGGTAATAGT		
		R: GTAGTGACAATAAACCTCCTA		
Tet(L)	267	F: TCGTTAGCGTGCTGTCATTC		
		R: GTATCCCACCAATGTAGCCG		
Tet(M)	159	F: AGTGGAGCGATTACAGAA		
		R: CATATGTCCTGGCGTGTCTA		
Tet(S)	589	F: ATCAAGATATTAAGGAC	58	
		R: TTCTCTATGTGGTAATC		
SulI	433	F: CGGCGTGGGCTACCTGAACG	65	Trimethoprim/Sulfamethoxazole
		R: GCCGATCGCGTGAAGTTCCG		
SulII	293	F: GCGCTCAAGGCAGATGGCATT	65	
		R: GCGTTTGATACCGGCACCCGT		

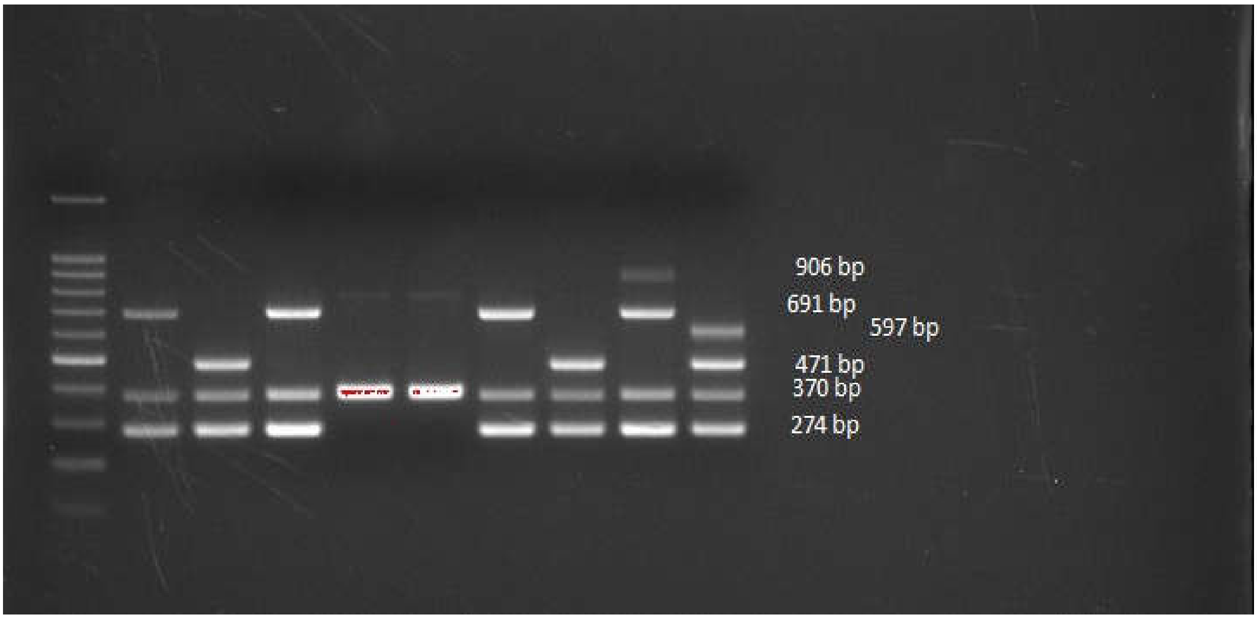

Figure S1: The electrophoretic profile of virulence genes detected in *Listeria monocytogenes* strains
